# CDC’s Public Health Surveillance of Cancer

**DOI:** 10.5888/pcd14.160480

**Published:** 2017-05-18

**Authors:** A. Blythe Ryerson, Greta M. Massetti

**Affiliations:** 1 Division of Cancer Prevention and Control, National Center for Chronic Disease Prevention and Health Promotion, Centers for Disease Control and Prevention, Atlanta, Georgia

## Abstract

Routine data collection efforts are a necessary, often underappreciated, component of nearly all cancer research and prevention efforts. Public health cancer surveillance data are crucial for identifying needs, planning interventions, directing public health resources, and evaluating the overall effectiveness of initiatives aimed at preventing or treating cancer and its negative health consequences. As the nation’s health protection agency, the Centers for Disease Control and Prevention (CDC) provides resources for disease surveillance systems to help protect our nation against expensive and dangerous health threats, including cancer. Therefore, public health surveillance is a core function of CDC. In this article we briefly describe CDC’s approach to cancer surveillance in our public health programs and other federal initiatives to monitor cancer-related outcomes. We also describe our premier cancer incidence surveillance system, the National Program of Cancer Registries, and discuss uses and applications of the program’s critical cancer data.

## CDC’s Public Health Surveillance of Cancer

Cancer is the second leading cause of death in the United States (1) and therefore a focus of public health efforts. One defining feature of the public health approach to cancer control is its reliance on population data for planning and evaluating efforts aimed at preventing the health consequences of cancer. The public health cancer prevention continuum (2,3) includes primary prevention such as tobacco control, human papilloma virus (HPV) vaccination, and removal of precancerous polyps during screening colonoscopies to prevent progression to colorectal cancer; secondary prevention (early detection) such as screening mammography and low-dose computed tomography among current or former heavy smokers to identify early-stage lung cancer; and tertiary prevention, which involves cancer survivorship care to reduce disability, other health outcomes associated with a cancer diagnosis or its treatment, and cancer recurrence and progression. Because of the heterogeneity of cancer, this prevention continuum differs across cancer types, as do the unique risk factors, associated disorders, and intervention strategies.

The World Health Organization defines public health surveillance as the continuous, systematic collection, analysis, and interpretation of health-related data needed for the planning, implementation, and evaluation of public health practice (4). This comprehensive definition of surveillance along with the heterogeneity of the disease and its complex public health prevention continuum means cancer surveillance is an exceptionally broad concept. It includes measurement of risk factors; assessment of use of health services such as vaccination rates and screening; and cancer prevalence, incidence, mortality, and survival rates and various outcomes affecting cancer survivors.

Conducting public health surveillance is one of CDC’s core functions, yet no comprehensive review of the agency’s heterogeneous public health cancer surveillance activities has been conducted. This article summarizes the key components of CDC’s cancer surveillance approach with a special emphasis on cancer incidence data collected through our National Program of Cancer Registries (NPCR). CDC compiles extensive data with direct and indirect implications for cancer, either collected directly or in collaboration with funded or unfunded partners. We focus on surveillance data used by CDC programs for planning, implementation, and evaluation of public health practice.

## CDC’s Approach to Cancer Surveillance

### Surveillance of cancer risk factors

CDC’s Division of Cancer Prevention and Control (DCPC) provides resources and technical expertise for cancer-related questions included in the Behavioral Risk Factor Surveillance System (BRFSS) (5). Questions are included in BRFSS on a routine, rotating basis related to breast cancer screening (mammography), cervical cancer screening (Papanicolaou test and HPV test), and colorectal cancer screening (home blood stool test, sigmoidoscopy, or colonoscopy). Cancer screening questions are included in BRFSS every 2 years in the core survey, which yields state-by-state prevalence and trends in cancer screening in accordance with the US Preventive Services Task Force (USPSTF) recommendations (6). DCPC also sponsors optional modules in support of CDC cancer program priorities, including questions on excess sun exposure (BRFSS 2010, 2012, and 2016 surveys), HPV testing and vaccination (BRFSS 2013 and 2014 surveys), shared decision making about prostate-specific antigen (PSA) tests (BRFSS 2012, 2013, 2015, and 2016 surveys), clinical breast examination (BRFSS 2015 and 2016 surveys), and cancer survivorship (BRFSS 2009, 2010, 2012, 2014, and 2016 surveys). Cancer survivorship questions include self-reported cancer diagnoses and basic treatment information, source of health care, health insurance issues, clinical trial participation, and other outcomes such as pain. The BRFSS core survey and other optional modules yield data on cancer risk factors such as smoking, obesity, and physical inactivity. BRFSS also collects information about comorbid conditions, which is critical given the large overlap between cancer and other chronic diseases (7). Information about which states administered which optional modules across years is available on the BRFSS website (www.cdc.gov/brfss/questionnaires/index.htm).

DCPC collaborates with the National Center for Health Statistics (NCHS) and the National Cancer Institute (NCI) to support questions and special supplements on the National Health Interview Survey (NHIS). The core cancer questions provide data on self-reported cancer prevalence, various cancer-associated health behaviors, and access to and use of cancer-related health. DCPC and NCI support the NHIS Cancer Control Supplement, which periodically presents a focused examination of issues pertaining to cancer-related behaviors, screening, and risk assessment. The cancer-related questions vary across years to reflect emerging needs. DCPC-sponsored NHIS questions have been asked in 2000, 2003, 2005, 2008, 2010, 2013, and 2015. More information about NHIS supplements is available on the NHIS website (www.cdc.gov/nchs/nhis/supplements_cosponsors.htm).

Other CDC data sources that routinely contribute to cancer surveillance and research include the National Health and Nutrition Examination Survey (www.cdc.gov/nchs/nhanes/index.htm), the Youth Risk Behavior Surveillance System (www.cdc.gov/healthyyouth/data/yrbs/index.htm), the Medical Expenditure Panel Survey (https://meps.ahrq.gov/mepsweb/), and program-specific clinical data collection initiatives supported through the National Breast and Cervical Cancer Early Detection Program (NBCCEDP) (8) and the Colorectal Cancer Control Program (9).

### Surveillance of cancer incidence and mortality

DCPC collects data on new cancer cases diagnosed each year through NPCR covering 96% of the United States (Figure) (10). When combined with data collected through NCI’s Surveillance, Epidemiology and End Results (SEER) program (11), these cancer data yield incidence information for 100% of the United States. These data collection efforts also provide estimates of cancer survival and prevalence. Cancer mortality data come from NCHS’s National Vital Statistics System (NVSS), which is the oldest and most comprehensive public health surveillance effort in the United States (12). Combining cancer incidence and mortality data provides CDC with one of the most sophisticated and complete public health surveillance systems in the nation.

**Figure:**
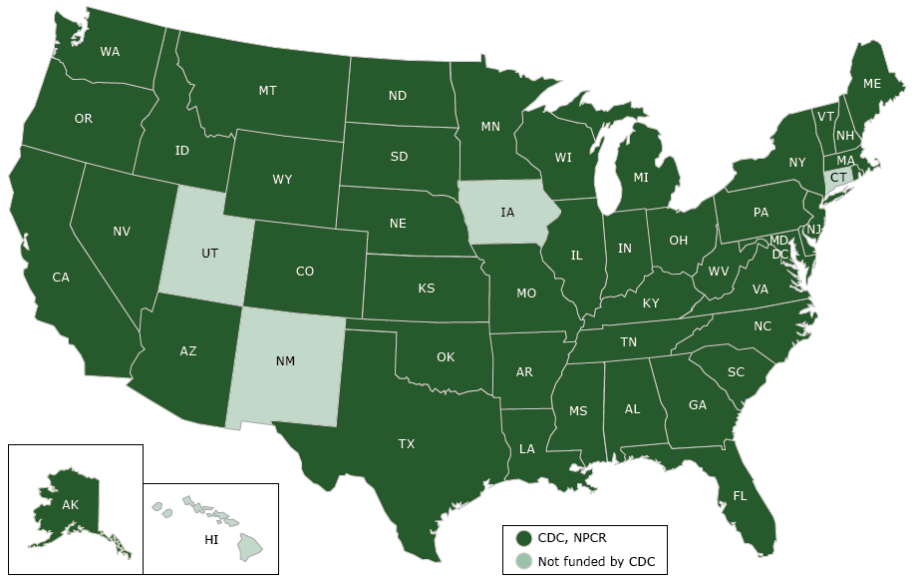
Centers for Disease Control and Prevention’s (CDC’s) National Program of Cancer Registries (NPCR) 2017 funding, showing which states receive funding from CDC’s NPCR and which do not.

## National Program of Cancer Registries (NPCR)

CDC’s cancer incidence data come from NPCR, which was established by Congress in 1992. From 2012 through 2017, CDC’s NPCR funded central cancer registries in 45 states, the District of Columbia, Puerto Rico, and the US Pacific Island jurisdictions. Central cancer registries are responsible for collecting, processing, and analyzing complex data from medical facilities on every in situ and invasive cancer (with the exception of basal cell and squamous cell carcinoma of the skin) diagnosed among residents in their geographic catchment area (eg, state). Consequently, data in the state or territorial central registries represent everyone diagnosed or treated for cancer. A brief summary of the data items collected by all central cancer registries is shown in Table 1. NPCR data collection occurs through medical record abstraction or electronic reporting at the health care facility level. Incident cases from all hospitals, physicians’ offices, pathology laboratories, treatment facilities, and other medical facilities are reportable to the central cancer registry.

**Table 1:** Summary of Data Items Collected by All Central Cancer Registries, United States

Data Type	Examples
Record identification	Registry identification
	Patient name^a^
Demographics	Patient address^a^
	Race
	Spanish/Hispanic origin
	Sex
	Date of birth
Cancer identification	Primary site
	Histologic type (ICD-O-3)
	Behavior code (ICD-O-3)
	Grade
	Date of diagnosis
Stage/prognostic factors	Stage
	Tumor size
Treatment	Type of first course of definitive treatment
	Date of first course of definitive treatment
Follow-up/recurrence/death	Date of last contact
	Date of death
	Underlying cause of death

Abbreviations: ICD-O-3, International Classification of Disease for Oncology, Third Edition.

a Centers for Disease Control and Prevention does not receive patient identification information.

The central cancer registry also links data with state and national databases to supplement and improve the quality of data. Every central cancer registry routinely links cancer data with that of its state death files, NBCCEDP, the National Death Index, and the Indian Health Service. Some registries optionally link with other data sources, such as Medicare and Medicaid claims files to address gaps identified in their data or improve the usefulness of the data for local cancer control activities. Because the data in a single record come from various sources, the central registry is responsible for consolidating the information into a single record on each cancer case and submitting a subset of its data to CDC annually, including any updates from previous years.

To ease the burden of such a complex data collection system, CDC has several surveillance informatics initiatives to automate processes and use electronic data exchange in cancer reporting. Examples include cancer reporting from ambulatory providers to registries for Stage 2 Meaningful Use and the development of a natural language processing web-based repository to facilitate analytic data extraction from text-based records (13,14).

NPCR uses North American Association of Central Cancer Registries uniform data standards (15) and works with NCI to ensure compatibility and comparability of cancer incidence and to publish annual data in the US Cancer Statistics Incidence and Mortality Web-based Report (16). Although this online report hosts the official US federal cancer statistics, NPCR and SEER data are also available through other online tools, including CDC-WONDER (https://wonder.cdc.gov/), CDC’s Environmental Public Health Tracking Network (www.cdc.gov/nceh/tracking/), and State Cancer Profiles (https://statecancerprofiles.cancer.gov/). NPCR analytic data sets are also available to researchers interested in using these data (https://www.cdc.gov/cancer/npcr/public-use/). Through careful coordination and collaborations with our cancer surveillance partners, NPCR data are comparable with those of other countries and are included in the Cancer Incidence in Five Continents (http://ci5.iarc.fr/Default.aspx) series providing information on the global burden of cancer.

## Application of Cancer Surveillance Data for Program Monitoring, Evaluation, and Research

Incidence data from NPCR and SEER and cancer mortality data from NCHS NVSS are the basis for nearly all cited cancer statistics and projections, including the Annual Report to the Nation on the Status of Cancer (17) and the American Cancer Society’s Cancer Facts and Figures (18). These surveillance activities, along with data collected through BRFSS, NHIS, and other initiatives, are vital to all cancer prevention programs.

### National Comprehensive Cancer Control Program

Established in 1998, the National Comprehensive Cancer Control Program (NCCCP) supports strategic approaches to preventing or minimizing the impact of cancer in communities (19). CDC funded comprehensive cancer control initiatives in all 50 states, the District of Columbia, 7 tribal groups, and 7 US Associated Pacific Islands and Territories from 2012 through 2017 to build and support coalitions of stakeholders, use data to define and monitor the cancer burden in their area, prioritize proven strategies for cancer control, develop cancer plans, and put cancer plans into action. NCCCP grantees develop state or local cancer plans based on the burden of disease defined through NPCR, SEER, and NVSS cancer incidence and mortality data. NCCCP grantees also use these data along with BRFSS risk factor, screening, and other cancer-related measures to evaluate and monitor cancer programs in their geographic regions. For example, cancer registry data have been used to show that Native Americans living in the Cherokee Nation have higher colorectal cancer death rates than do other Native Americans living in Oklahoma and across the United States. This information has been used by the Cherokee Nation Comprehensive Cancer Control Program to engage the Prevent Cancer Foundation to educate and empower Cherokee Nation citizens in preventing and controlling colorectal cancer (20).

### National Breast and Cervical Cancer Early Detection Program

The NBCCEDP is a nationwide, comprehensive public health program that provides uninsured and underserved women access to timely breast and cervical cancer screening and diagnostic services (8). The NBCCEDP is implemented through cooperative agreements between CDC and all 50 states, the District of Columbia, 5 US territories, and 11 American Indian/Alaska Native tribes or tribal organizations and has been implemented from 2012 through 2017. In addition to providing direct services to women, grantees are required to collect and report a set of standardized data elements for monitoring client demographics and clinical outcomes of women screened through the NBCCEDP. These data are also used to establish program policies and practices, assess the national program’s screening outcomes, and respond to information needs of CDC stakeholders and partners. NBCCEDP data are routinely linked by grantees to cancer registries to confirm diagnosis and cancer prognostic factors and to allow comparisons of cancer incidence in program and nonprogram populations. A recent analysis (2015) used these data to compare characteristics and cancer stage distribution of women enrolled with women not enrolled in NBCCEDP (21). Findings from that analysis suggest that the program has been effective in achieving its goal of enrolling racial and ethnic minority populations.

### Colorectal Cancer Control Program

CDC’s Colorectal Cancer Control Program (CRCCP) helps states and tribes across the United States increase colorectal cancer screening rates among men and women aged 50 to 75 years (9). The program focuses on increasing screening by implementing priority evidence-based interventions recommended by the *Guide to Community Preventive Services*. This approach allows grantees to implement targeted evidence-based interventions and supporting strategies on a feasible scale and collect program-level and patient-specific clinical data that show the program’s impact. In 2015, CRCCP supported 24 state health departments, 6 universities, and 1 American Indian tribe. Six grantees were also funded to provide screening to uninsured and underinsured people; as in the NBCCEDP, these 6 grantees also report standardized clinical data for people screened.

### Healthy People 2020 and other national objectives

Healthy People 2020 (HP2020) (www.healthypeople.gov/) provides quantifiable national objectives for improving the health of all Americans. For cancer, HP2020 aims to reduce the number of new cancer cases and the illness, disability, and death caused by cancer. The 27 cancer objectives support monitoring trends in incidence, death, and survival and reflect the importance of promoting evidence-based screening for cervical, colorectal, and breast cancer by measuring the use of screening tests recommended by the USPSTF. The cancer-related HP2020 objectives are measured through various CDC-supported data systems (Table 2).

**Table 2:** Healthy People 2020 Objectives and Associated Data Sources Related to Cancer, United States

Objective	Data Source
Reduce the overall cancer death rate	NVSS-M
Reduce the lung cancer death rate	NVSS-M
Reduce the female breast cancer death rate	NVSS-M
Reduce the death rate from cancer of the uterine cervix	NVSS-M
Reduce the colorectal cancer death rate	NVSS-M
Reduce the oropharyngeal cancer death rate	NVSS-M
Reduce the prostate cancer death rate	NVSS-M
Reduce the melanoma cancer death rate	NVSS-M
Reduce invasive colorectal cancer	NPCR, SEER
Reduce invasive uterine cervical cancer	NPCR, SEER
Reduce late-stage female breast cancer	NPCR, SEER
Increase the number of central, population-based registries from the 50 states and the District of Columbia that capture case information on at least 95%of the expected number of reportable cancers	NPCR, SEER
Increase the proportion of cancer survivors who are living 5 years or longer after diagnosis	SEER
Increase the mental and physical health-related quality of life of cancer survivors (developmental objective)	NHIS^a^
Increase the proportion of women who receive a cervical cancer screening based on the most recent guidelines	NHIS
Increase the proportion of adults who receive a colorectal cancer screening based on the most recent guidelines	NHIS
Increase the proportion of women who receive a breast cancer screening based on the most recent guidelines	NHIS
Increase the proportion of women who were counseled by their providers about mammograms	NHIS
Increase the proportion of women who were counseled by their providers about Papanicolaou (Pap) tests	NHIS
Increase the proportion of adults who were counseled by their providers about colorectal cancer screening (developmental objective)	NHIS^a^
Increase the proportion of men who have discussed the advantages and disadvantages of the prostate-specific antigen (PSA) test to screening for prostate cancer with their health care provider	NHIS
Reduce the proportion of adolescents in grades 9 through 12 who report sunburn (developmental objective)	YRBSS^a^
Reduce the proportion of adults aged 18 years or older who report sunburn	NHIS
Reduce the proportion of adolescents in grades 9 through 12 who report using artificial sources of ultraviolet light for tanning	YRBSS
Reduce the proportion of adults aged 18 or older who report using artificial sources of ultraviolet light for tanning	NHIS
Increase the proportion of adolescents in grades 9 through 12 who follow protective measures that may reduce the risk of skin cancer	YRBSS
Increase the proportion of adults aged 18 years and older who follow protective measures that may reduce the risk of skin cancer	NHIS

Abbreviations: NHIS, National Health Interview Survey; NPCR, National Program of Cancer Registries; NVSS-M, National Vital Statistics System-Mortality; SEER, Surveillance, Epidemiology and End Results; YRBSS, Youth Risk Behavior Surveillance System.

a Potential data source for a developmental objective. Data sources were available for baseline but may not be available for monitoring changes.

### Research

Several projects have used central cancer registries to recruit participants for research studies. Population-based cancer registries are useful in identifying samples of participants who meet criteria for certain cancers. For example, one study assessed health-related quality of life and health behaviors among colorectal cancer survivors recruited through the California Cancer Registry demonstrating that the cancer registry can be a valuable source for identifying and recruiting study participants (22,23). Cancer registries have also been used to conduct laboratory studies, such as a study that used tissue from HPV-related cancers from 7 central cancer registries. Researchers were able to assess the proportion of HPV-related cancers attributable to different types of HPV and estimate the potential impact of scaling up coverage of HPV vaccines (24). Other uses of NPCR data have involved comparative effectiveness research applications by enhancing data collection to address targeted questions, including the clinical use and prognostic value of specific biomarkers (25). This enhanced data collection has leveraged NPCR as a resource to conduct health services research and examine patterns and disparities in cancer care.

## Future Directions

DCPC is committed to continuously improving the accuracy, efficiency, and timeliness of all cancer surveillance efforts it directly funds or supports. One way cancer registries are doing this is by remaining on the cutting edge of health information technology by facilitating more electronic reporting and automated data management techniques. Through work with the cancer registry community and other key partners, such as the Office of the National Coordinator and the Centers for Medicare & Medicaid Services, NPCR has made great progress in developing a robust capability for electronic exchange of certain cancer data. However, there is much work to do. Sustained local, state, and federal cancer registry commitments are needed to continue to develop new tools and software for the development and acceptance of additional electronic cancer records. Through these efforts, the cost-effectiveness and timeliness of cancer surveillance is expected to improve in the long term as registry staff spend less time engaged in visual review, coding, and data entry and more time in quality assurance to improve the quality, completeness, and timeliness of the data. CDC’s NPCR will continue to build the necessary infrastructure for electronic reporting so that these efficiencies can be realized in the future. Cancer surveillance data are the core of cancer epidemiology and outcomes in clinical cancer research. CDC is dedicated to remaining a diligent steward of population-based cancer surveillance and maintaining complete capabilities in cancer incidence surveillance throughout the entire nation to support state and local activities. We also remain steadfast in making all CDC data useful and available to those who need them, particularly for program planning and monitoring, resource allocation, and state and federal accountability.

## References

[1] Centers for Disease Control and Prevention. Leading causes of death. http://www.cdc.gov/nchs/fastats/leading-causes-of-death.htm. Accessed July 26, 2016.

[2] Baumann L , Karel A . Prevention: primary, secondary, tertiary. Encyclopedia of behavioral medicine. New York (NY): Springer Science+Business Media; 2013. p. 1532-4.

[3] National Cancer Institute. Cancer control continuum. .http://cancercontrol.cancer.gov/od/continuum.html. Accessed July 26, 2016.

[4] World Health Organization. Public health surveillance. http://www.who.int/topics/public_health_surveillance/en/. Accessed July 26, 2016.

[5] Centers for Disease Control and Prevention. Behavioral Risk Factor Surveillance System [updated July 26, 2017]. http://www.cdc.gov/brfss/index.html. Accessed July 26, 2016.

[6] US Preventive Services Task Force. Recommendations for primary care practice. http://www.uspreventiveservicestaskforce.org/Page/Name/recommendations. Accessed July 26, 2016.

[7] Koene RJ , Prizment AE , Blaes A , Konety SH . Shared risk factors in cardiovascular disease and cancer. Circulation 2016;133(11):1104–14. 10.1161/CIRCULATIONAHA.115.020406 26976915 PMC4800750

[8] Centers for Disease Control and Prevention. National Breast and Cervical Cancer Early Detection Program. http://www.cdc.gov/cancer/nbccedp/index.htm. Accessed July 26, 2016.

[9] Centers for Disease Control and Prevention. Colorectal Cancer Control Program. http://www.cdc.gov/cancer/crccp/index.htm. Accessed July 26, 2016.

[10] Centers for Disease Control and Prevention. National Program of Cancer Registries. http://www.cdc.gov/cancer/npcr/index.htm. Accessed July 26, 2016.

[11] National Cancer Institute. Surveillance, Epidemiology and End Results Program. http://seer.cancer.gov/. Accessed July 26, 2016.

[12] Centers for Disease Control and Prevention. National Vital Statistics System. http://www.cdc.gov/nchs/nvss/index.htm. Accessed July 26, 2016.

[13] Centers for Disease Control and Prevention. National Program of Canter Registries, meaningful use of electronic health records. https://www.cdc.gov/cancer/npcr/meaningful_use.htm. Accessed July 26, 2016.

[14] Centers for Disease Control and Prevention. National Program of Cancer Registries, Registry Operations Resources (Informatics) [updated December 13, 2016]. https://www.cdc.gov/cancer/npcr/informatics/index.htm. Accessed December 13, 2016

[15] North American Association of Central Cancer Registries. Data standards and data dictionary (Volume II). http://www.naaccr.org/StandardsandRegistryOperations/VolumeII.aspx#. Accessed July 27, 2016.

[16] United States Cancer Statistics. 1999–2013 Incidence and mortality web-based report. US Department of Health and Human Services, Centers for Disease Control and Prevention, and National Cancer Institute; 2016. https://nccd.cdc.gov/uscs/. Accessed July 27, 2016.

[17] Ryerson AB , Eheman CR , Altekruse SF , Ward JW , Jemal A , Sherman RL , Annual report to the nation on the status of cancer, 1975–2012, featuring the increasing incidence of liver cancer. Cancer 2016;122(9):1312–37. 10.1002/cncr.29936 26959385 PMC4840031

[18] American Cancer Society. Cancer facts and figures 2016. Atlanta (GA): American Cancer Society; 2016.

[19] Centers for Disease Control and Prevention. National Comprehensive Cancer Control Program. http://www.cdc.gov/cancer/ncccp/index.htm. Accessed July 27, 2016.

[20] Centers for Disease Control and Prevention. Stories of success: National Comprehensive Cancer Control Program comprehensive cancer control in action. Atlanta (GA): Centers for Disease Control and Prevention, Division of Cancer Prevention and Control; 2010 http://www.cdc.gov/cancer/ncccp/pdf/success/successstories.pdf. Accessed July 27, 2016.

[21] Wu M , Austin H , Eheman CR , Myles Z , Miller J , Royalty J , A comparative analysis of breast cancer stage between women enrolled in the National Breast and Cervical Cancer Early Detection Program and women not participating in the program. Cancer Causes Control 2015;26(5):751–8. 10.1007/s10552-015-0548-x 25761406 PMC4612348

[22] Hawkes AL , Pakenham KI , Chambers SK , Patrao TA , Courneya KS . Effects of a multiple health behavior change intervention for colorectal cancer survivors on psychosocial outcomes and quality of life: a randomized controlled trial. Ann Behav Med 2014;48(3):359–70. 10.1007/s12160-014-9610-2 24722960

[23] Rodriguez JL , Hawkins NA , Berkowitz Z , Li C . Factors associated with health-related quality of life among colorectal cancer survivors. Am J Prev Med 2015;49(6, Suppl 5):S518–27. 10.1016/j.amepre.2015.08.007 26590647 PMC6334761

[24] Saraiya M , Unger ER , Thompson TD , Lynch CF , Hernandez BY , Lyu CW , ; HPV Typing of Cancers Workgroup. US assessment of HPV types in cancers: implications for current and 9-valent HPV vaccines. J Natl Cancer Inst 2015;107(6):djv086. 10.1093/jnci/djv086 25925419 PMC4838063

[25] Chen VW , Eheman CR , Johnson CJ , Hernandez MN , Rousseau D , Styles TS , Enhancing cancer registry data for comparative effectiveness research (CER) project: overview and methodology. J Registry Manag 2014;41(3):103–12. 25419602 PMC4524450

[26] US Department of Health and Human Services, Office of Disease Prevention and Health Promotion. Healthy People 2020. https://www.healthypeople.gov/. Accessed July 26, 2016.

